# Queer Space: Toilet Provision, Access and Inclusion in the West Midlands

**DOI:** 10.1007/s13178-021-00658-8

**Published:** 2021-10-25

**Authors:** Ben Colliver, Melindy Duffus

**Affiliations:** Birmingham City University, Curzon Building, 4 Cardigan Street, Birmingham, B4 7BD UK

**Keywords:** Gender, Sexuality, Space, Toilets, Inclusion, Access, Exclusion

## Abstract

**Introduction:**

This article explores issues relating to toilet provision in queer spaces. With a specific focus on the implementation of gender neutral toilets, it interrogates both practical and symbolic issues of inclusivity and accessibility.

**Methods:**

The findings presented in this paper are based on 12 semi-structured interviews that were conducted and analysed in 2020. The data was analysed thematically, utilising an inductive approach to analysis.

**Results:**

The results from this study highlight that spaces often considered ‘inclusionary’ operate within a number of ‘exclusionary’ frameworks. These unspoken and informal ‘rules’ and practices operate to exclude people considered ‘undesirable’ and function to uphold power structures that privilege cisgender, white gay men.

**Conclusions:**

This article extends our understanding of the ways in which people engage with, and access, both gender neutral and sex-segregated toilets. Through an analysis of complex issues relating to accessibility, inclusivity and the politicisation of queer spaces, this article argues that the implementation of gender neutral toilets holds strong practical and symbolic power within a heteronormative, cis-normative society.

**Policy Implications:**

The results from this study indicate that providing more gender neutral spaces improves accessibility for a range of people, but also has significant political power in challenging cis-normative, heteronormative standards.

## Introduction

In England and Wales, transgender people have been legally entitled to use gender-segregated toilets that align with their gender identity since the introduction of the Equality Act (2010). However, these rights have been subject to social and media attention, particularly in relation to toilet provision for children and young people (Pasha-Robinson, 2016). The term ‘transgender’ is used throughout this article to describe people who do not identify with the sex they were assigned at birth based on observation of perceived biological sex. The term transgender incorporates a range of identities that do not conform to expected gender norms and therefore functions as an umbrella term (Englert & Dinkins, 2016). However, whilst we use this term, it is important to acknowledge that this is not a universally accepted term and has long been subject to debate and dissent within parts of the communities that are incorporated under it, with it being highlighted that it fails to capture the nuances, diversities and needs of those communities (Monro, 2003). We also use the term ‘cisgender’ throughout this article when referring to those whose gender identity is consistent with the sex they were assigned at birth (Stryker, 2008). Whilst some people across social media actively reject the label of cisgender, research often identifies people as transgender, whilst allowing cisgender identities to remain the unspoken norm (Johnson, 2015). This reinforces the remarkability of transgender identities and allows for the interrogation and ‘othering’ of transgender people whilst presenting cisgender identities as unremarkable (Rumens, 2018). For those reasons, we use both terms throughout, in order challenge dominant narratives that position cisgender identities as not in need of identification. We also draw upon the term ‘non-binary’ throughout this article to describe individuals whose gender identity is between or beyond stable, hegemonic and normative categories of male and/or female (Richards et al., 2016). The term may also be used as an umbrella term to include gender identities including gender-queer, gender-fluid and bi-gender, although this is not an exhaustive list (Vijlbrief et al., 2020). Their gender identity may be identified between masculine and feminine, or there may also be a partial or complete rejection of the gender binary (Monro, 2019).

In April 2021, the Department of Education released guidance to schools in Ireland allowing the introduction of ‘gender neutral’ toilet provision within schools (O’Loughlin, 2021). Whilst inclusive toilet provision has been argued to benefit various groups of people, in recent years, public toilets have garnered significant social attention, becoming centred in discussions around transgender people (Jones & Slater, 2020). Discussions often framed as ‘debate’ have occurred on social media platforms extensively concerning social and legal entitlements to self-declare gender and the rights this provides to transgender people. Whilst trans equality is a human rights issue, online discussion is often one-sided, framed as a ‘debate’, whilst often silencing and excluding trans people from participation in these discussions. Self-declaration is often a contentious issue and often understood in a range of ways. In this context, self-declaration relates to an individual’s autonomy to declare their gender, making it easier for transgender people to achieve legal recognition of their self-declared gender without necessarily having met all of the criteria specified in the 2004 Act (Government Equalities Office, 2018). However, these ‘debates’ have also played out internationally in policy and practice. Notably, in 2017, President Donald Trump overturned instructions previously issued in 2016 by then-President Barack Obama that directed schools across the USA to allow students to access toilets consistent with their gender identity. Since then, 16 states have proposed or considered legislation that would restrict people to only using public, sex-segregated facilities consistent with their ‘observed’ sex at birth (Myers, 2018).

In a UK context, discussions around access to these spaces were fuelled by the 2018 government consultation on reforming the 2004 Gender Recognition Act which enabled people who had been diagnosed with ‘gender dysphoria’ to change their gender legally by obtaining a gender recognition certificate. This required approval from a ‘gender recognition panel’ consisting of legal and medical professionals to ensure that all necessary criteria had been met. The proposed reform of the Gender Recognition Act aimed to streamline this process and enable transgender people to obtain a gender recognition certificate without necessarily having met all of the criteria specified in the initial act (Government Equalities Office, 2018). This resulted in various discussions occurring around the implications this may have for women and children’s safety. These discussions often occurred in online spaces and focused on the potential for cisgender men to gain access to women and children in order to sexually offend, although some discussion also focused on the inherent ‘sexual deviance’ of transgender people (Colliver & Coyle, 2020). Despite this, other countries including Denmark and Ireland, have already implemented legislation that recognises gender as a self-declared category. This is specifically relevant to the West Midlands, which became a focal point for school protests around LGBTQ-inclusive education, creating a culture of exclusion for LGBTQ people (Colliver, 2018).

Transgender communities have attracted significant political, social and academic attention in recent years, with an increasing body of research exploring issues relating to, and affecting, transgender people (Colliver, 2021a; Pearce, 2018; Shuster, 2017). However, at the same time, transgender identities have been subject to significant discussion and de-legitimisation across social media platforms, in which the authenticity of ‘transgender’ as an identity category has been questioned and interrogated (Colliver et al., 2019). Whilst transgender people are not a new phenomenon and have historically existed across societies globally (Jamel, 2018), it is only more recently, within the UK, that such intense levels of public scrutiny have occurred (Colliver et al., 2019). The visibility afforded to transgender people as a result of legislative developments, media representations and social media has resulted in the lives of transgender people being thrust into public discussions. At the same time, England and Wales have continued to see an annual incline in the amount of police-recorded hate crimes against transgender people (Home Office, 2020), although it is likely that this is an underrepresentation of how much transphobic hate crime actually occurs, as it is argued that hate crimes are substantially underreported (Lombardi et al., 2008), and this may be for a number of reasons including a mistrust of the police, the prevalence of hate and a perception that the police will be unable to help.

The first part of this article explores existing debates around public toilet provision to interrogate its inclusivity in ‘queer spaces’. We situate these debates within feminist discourse. Next, the methodological approach used in this study is examined before reporting key findings. The findings denoted three central themes. First, the ways in which heterosexual, queer, cisgender and transgender people engage with toilet provision in queer spaces. This article demonstrates how hetero- and cis-normative privilege influences the ways in which heterosexual and cisgender people understand gender neutral toilets within queer spaces. The discomfort with gender neutral toilets in queer spaces experienced by heterosexual, cisgender people reflects privilege in which their needs and norms are met through heteronormative social norms. It is not the work of queer spaces to accommodate heterosexual, cisgender comfort. Second, how the way that gender neutral toilets are positioned as an explicitly ‘queer’ issue negates the need for any progression or adaptation to be made outside of ‘queer spaces’. Third, the ways in which the materiality and location of toilets within queer spaces work to exclude particular groups and cater primarily for men. These contributions are significant to sociological thought in advancing understanding of the contextual and spatial challenges of navigating and engaging in queer spaces.

## Public Toilets, Inclusion and Gender Policing

It has been argued that public toilets become the focal point of discussions around gender as they represent the ultimate sex-segregated spaces and therefore represent a site of contention for those wishing to enforce sex segregation (Doan, 2010; Greed, 2019). Existing research has shown transgender people often experience gender policing, hostility and exclusion when accessing sex-segregated spaces (Colliver, 2021b). The same research also highlighted the benefits of gender neutral toilets as they relate to feelings of inclusion and safety, particularly for individuals who are non-binary. Gender neutral toilets can be constructed in a range of different ways as physical spaces. Ideally, these would consist of purpose-built, custom-made, self-contained cubicles which provide individual privacy to users. This type of gender neutral toilet offers floor-to-ceiling walls and doors and may contain individual washing facilities, although often, washing facilities are communal. However, in the development of gender neutral toilets, a ‘quick fix’ approach has often been adopted, which usually takes one of two approaches. Firstly, existing accessible toilets, which are usually single occupancy, have had signage changed to indicate that they are now ‘gender neutral’ spaces. This approach has been criticised for reducing the availability of toilets that are physically accessible (Ramster et al., 2018). The second approach involves changing signage on existing sex-segregated toilets, usually women’s toilets due to the presence of urinals in men’s toilets, and designating them as gender neutral without any modification. This approach has also attracted critique, highlighting historical concerns about disproportionate toilet provision for men and women. It has been argued that men have two-thirds greater toilet provision than women in public spaces (Anthony & Dufresne, 2007). Concerns have been raised that the reclassification of women’s toilets as gender neutral results in increased usage of what was previously women’s toilet provision, thereby exacerbating the relative lack of provision for women (Greed & Daniels, 2002).

Public toilets have been argued to be a site of significant risk and concern for transgender people (Faktor, 2011). This has been framed by Doan (2010, 635) as a result of transgender people experiencing a ‘special kind of tyranny – the tyranny of gender – that arises when people dare to challenge the hegemonic expectations for appropriately gendered behaviour in Western society’. It is important to note that hegemonic expectations of gender are spatially and culturally dependent and therefore the meanings attached to bodies and gendered embodiment are historically and spatially located (Longhurst, 2005). Resultantly, the ways in which transgender and gender non-conforming people present their gendered identities vary across spaces and contexts and may change in order to reduce the risk of experiencing violence, hostility and prejudice as a result of ‘gender-policing’. As Nash (2010, 588) notes, bodies and spaces ‘simultaneously (re)create one another’, and the spaces we occupy have material consequences for how we traverse them.

The tyranny of gender may therefore be most forcefully experienced within sex-segregated spaces that allow for others to interpret our gendered identities and to apply and impose heteronormative, cis-normative expectations of gender presentation. Sex-segregated toilets are differentiated by constructions of biological distinctions between men and women and have therefore been described as ‘sites where individuals’ bodies are continually policed and (re)placed within sex categories’ (Browne, 2004, 332–3). As Cavanagh (2010, 4) argues, ‘nowhere are the signifiers of gender more painfully acute and subject to surveillance than in sex-segregated washrooms’. This may be further heightened by the spatial construction of public toilets, often illuminated with mirrors, which facilitates surveillance within these spaces (Bender-Baird, 2016; Cavanagh, 2010). Violence, discrimination and abuse may therefore occur within these spaces resulting from a discontinuity between an individual’s gender identity and how others interpret their gender presentation. This is what Browne (2004, 332) has identified as ‘genderism’, which describes ‘hostile readings of gender ambiguous bodies’. Heightened levels of gender surveillance can therefore legitimise violence and abuse against transgender and gender non-conforming people (Namaste, 2000) who are perceived as challenging “the ‘natural’ connections between sexed embodiments and sexed lives” (Browne, 2004, 333). Safe access to public toilets has material implications for people in relation to their ability to engage in various aspects of social life. However, given the considerations made so far, it is unsurprising that public toilets represent spaces of policing and abuse for transgender people (Browne, 2004; Doan, 2010; Faktor, 2011).

Whilst significant ‘debate’ has occurred regarding trans women’s access to public toilets, existing research shows that a lack of gender neutral toilet provision may be problematic for non-binary people (Paechter et al., 2021). This may result in non-binary individuals travelling outside of a particular venue to access gender neutral toilets (Paechter et al., 2021). Similar to some binary-trans people, non-binary people have reported feeling policed within binary gendered spaces (Bower-Brown et al., 2021). However, access to these facilities is not a priority for all non-binary people, with some reluctant to occupy these spaces for fear of being ‘outed’ (Paechter et al., 2021). Therefore, it is key to consider the ways in which non-binary people occupy and access these spaces, as most research has focused on provisions within schools, rather than within spaces deemed to be ‘queer’.

### Feminist Exclusion, Queer Inclusion?

When exploring issues and contestations around the implementation of gender neutral toilets, it is not only the practical issues outlined previously that are drawn upon to maintain the status quo of sex segregation. Rather, access and inclusion within public toilets have been centred as a feminist issue. Transgender inclusion has created divisions within feminist movements with a binary trade-off being established that positions ‘transgender rights’ against ‘women’s sex-based rights’. This draws a distinction between an apparent ‘biological reality’ and ‘social ideology’ (Pearce et al., 2020). ‘Gender critical’ feminists who are perceived as advocating for the exclusion of transgender people, most notably transgender women, from sex-segregated spaces are commonly referred to as TERF’s (trans-exclusionary radical feminists), originally utilised by some cisgender women to differentiate their radical feminist principles from other trans-exclusionary perspectives (Smythe, 2018). However, many now claim that TERF is pejorative and describe themselves as ‘gender critical’ (see Pearce et al., 2020 for a more detailed overview).

One of the central arguments put forward by ‘gender critical’ feminists for the maintenance of sex-segregated toilets, and also the exclusion of transgender women from women’s toilets focuses on the risk of sexual violence. Research has shown that women may experience disproportionate levels of fear in public spaces, and this fear primarily concerns the risk of physical and sexual violence (Pain, 1997; Vera-Gray & Kelly, 2020). However, the gendered nature of fear has often been considered contradictory as women experience less victimisation than men, but experience higher levels of concern about victimisation (Hale, 1996). This has been challenged on the basis that experiences of sexual harassment are routinely excluded from victimisation surveys, and therefore the actual rate of women’s victimisation is significantly underrepresented (Vera-Gray & Kelly, 2020). It is also important to note that the fear of victimisation is spatially contextual, with urban public spaces and spaces with high populations of male strangers are most closely associated with fear of victimisation (Doran & Burgess, 2011; Yeoh & Yoew, 1997). Resultantly, gender neutral toilets may be perceived as sites of fear for all women in relation to physical and sexual violence; however, gender critical perspectives often centre the fear of cisgender women. Indeed, online discourse around risk of sexual violence in public toilets has certainly positioned cisgender women as at risk of male violence, ignoring the risk posed to transgender women (Colliver & Coyle, 2020).

A heightened sense of vulnerability to physical and sexual violence may be a result of individuals finding themselves in various states of undress; however, this is likely to be context dependent. Women’s toilets within the night-time economy are often considered to be a ‘safe space’ for women where they can obtain physical distance from men in the wider space and avoid the ‘male, heterosexual gaze’ (Browne, 2004). The dynamics of women’s toilets may offer respite and social opportunities within a male-dominated public sphere in which women can engage in women-only social interactions, rest and show emotion (Ramster et al., 2018). As Jones and Slater (2020, 835) argue, “for many ‘gender-critical’ feminists, the walls of women-only facilities have come to symbolise the boundaries of womanhood: a ‘safe’ space where the terms of inclusion are vehemently regulated and protected”. Therefore, some objections to toilet desegregation may stem from a perceived loss of a ‘safe space’ for women.

On the other hand, queer spaces have often been associated with subverting hegemonic gendered expectations (Bailey, 2013). We use the term ‘queer spaces’, although acknowledge these had historically and predominantly been referred to as ‘gay spaces’. However, it is not all queer spaces that are associated with subverting gender norms, as Hale and Ojeda (2018) argue that dominant heteronormative configurations of masculinity, particularly in white, gay male cultures prevent the liberation of masculinity, and therefore opposing femininity is a central part of ‘belonging’. Issues of misogyny within queer spaces have been documented, interrogating the complex relationship between masculinities, queer spaces and femininities (Colliver, 2021a; Hale & Ojeda, 2018). Despite queer spaces and queer culture often being considered oppositional to societal norms (Warner, 1993), characterised through the celebration of ‘deviant’ expressions of gender and sexuality, these spaces often reinforce the gender binary through the provision of sex-segregated toilets.

As space is socially constructed, it is key to acknowledge that an individual’s age, ethnicity, gender, sexuality, class and disabilities influence whether someone can access particular social spaces, and how these spaces are navigated (England, 2018). As Koskela (1999) argues, space is not simply an area for interaction, but is simultaneously reproduced by these interactions. Speaking specifically to gender, Massey (1994, 179) argues that ‘space and place are important in the construction of gender relations’ as a result of the symbolic meanings they convey. Therefore, the spatial organisations of venue’s has material consequences for the level of inclusion available for socially marginalised groups. It is also important to note that, in the UK, a significant amount of metropolitan ‘queer spaces’ may be characterised by ‘homonormativity’ (Brown, 2012). Little has been done to explore the ways that ‘homonormative’ identities (Podmore, 2013) are produced and normalised within mainstream LGBTQ venues. Homonormativity can be described as the normalisation of certain queer identities, which are usually cisgender, white gay men which are consumable and assimilated into heteronormative ideals and expectations of what ‘queer’ looks like. Resultantly, queer people who do not conform to these idealised standards may experience exclusion and othering from ‘mainstream’ queer venues. Indeed, as Namaste (2000) argues, transgender people may be at risk of discrimination and violence in both heteronormative public spaces, but also in those signified to be queer. In this article, we address some of these issues through a detailed exploration of toilet provision within queer spaces in the West Midlands.

## Methodology

Empirical data has been reported from 12 semi-structured interviews conducted by both authors with individuals aged 18 or over, who have visited a ‘gay village^1^’ within the West Midlands in the UK. Data was collected and analysed throughout 2020. The interviews formed part of a larger research project that also involved direct observations, conducted by both researchers over a 3-month period of 4 venues within the ‘gay village’ in the West Midlands. The larger research project sought to explore experiences of inclusion and exclusion within these queer spaces, interrogating how normative identities that ‘belong’ are established, and how others may be positioned as ‘outsiders’, along lines of race, gender, disabilities and age. In recent years, there has been a noticeable level of ‘othering’ towards the LGBTQ community within the West Midlands. This may be most noticeable through the intense school protests that occurred in Birmingham in response to LGBTQ inclusive education (BBC News, 2019). These types of experiences and views enforce issues of exclusion, with the LGBTQ community being seen more as sinful, than a legitimate part of the community. Despite this overarching othering of the LGBTQ community, there appears to be further ‘othering’ occurring within the community itself. This is of particular importance, as with more people acknowledging and recognising gender and sexual non-conformity; this enhances the level of diversity within the LGBTQ community and as such of who is entering these spaces. With this in mind, it is necessary that policy and practice initiatives support the development of inclusive and welcoming communities. The study aimed to develop outcomes that help in the promotion of inclusive practice within LGBTQ venues within the West Midlands.

For the purpose of this article, we focus solely on the data elicited from the semi-structured interviews. This study is exploratory in nature, adopting a case study approach, utilising a specific geographic area to elicit a deeper understanding of the topic. The focus of the interviews was on participants’ experiences of accessing and navigating queer spaces within the West Midlands. Although queer spaces may take many different forms, we particularly focused on spaces associated with the night-time economy, and as such, participants were asked about their experiences within bars, pubs and clubs within the West Midlands that are known to be ‘LGBTQ’ spaces. Although the night-time economy may encompass non-alcohol-centred leisure spaces (Shaw, 2014), the gay village which was the focus of this research is highly associated with alcohol consumption. These spaces are deemed to be ‘LGBTQ’ spaces through community interaction and access or the venue advertising itself as such. Participants were asked to share their experiences of occupying and navigating these spaces, with a particular focus on their identity and issues of access, inclusion and exclusion. Whilst toilet provision within these spaces was not a direct question, it became central in participants’ narratives of their experiences within these spaces.

Purposive sampling was utilised for this research project, as we were specifically seeking participants aged 18 or over, who had visited the ‘gay village’ within the West Midlands in the last 12 months. It is worth noting that these individuals will have visited the ‘gay village’ prior to the March 2020 lockdown, and as such minimised or removed the chance of participants discussing experiences of COVID-19 restrictions within the ‘gay village’. It is also important to acknowledge that only people who had accessed these spaces were included in this project and therefore have navigated these spaces in some way. However, it does mean that individuals who have visited these spaces previously, but not within the last 12 months, were not included. This may therefore mean that the views of people who have never accessed these spaces have not been collected, and therefore, future research may seek to be more inclusive in its recruitment criteria. Participants were primarily recruited through social media, which although has limitations relating to the representativeness of any given sample proved to be the most effective method of reaching out to participants. The advantage of this approach is that it enables the participant to have the relevant knowledge of the topic area (Denscombe, 2010) and provides flexibility to enable all relevant topic areas to be discussed, whilst providing opportunity for development (Adams, 2015). A relatively diverse sample was recruited in relation to gender, race, religion, disability status and sexuality. When considering the diversity of the sample, it was made up of 5 male participants, 5 female participants and 2 non-binary participants and of the sample 4 participants identified as transgender. Whilst 6 of 12 participants identified as white British, 2 identified as black British, 2 identified as Asian British, 1 identified as white European, and 1 identified as mixed heritage. Most participants were non-religious; however, 2 participants are Christian, 1 participant is Muslim, and 1 participant is Sikh. Participants ages ranged from 18 to 52, with most participants falling between the ages of 21 and 40. Only four participants declared as having a disability, and these were a range of learning disabilities, sensory disabilities and physical disabilities. Finally, in relation to sexuality, 5 of the participants identified as heterosexual, 4 described themselves as gay, 1 described themselves as a lesbian, 1 identified as queer and 1 identified as pansexual. Participants of any sexuality were recruited in this study as although the focus was on queer spaces, it was acknowledged that these spaces are accessed by heterosexual, cisgender people as well. It was also recognised that individuals may be heterosexual in relation to their sexuality, but may still consider themselves queer in relation to their gender identity and/or expression.

The semi-structured interviews were transcribed verbatim, and interviews were fully transcribed. The data was analysed thematically, guided by the six steps outlined by Braun and Clarke (2006). An inductive approach was taken to analyse the data as the lack of current research around how people engage with toilets in queer spaces created difficulty in trying to locate pre-existing themes (Saldana, 2013). However, in line with Braun and Clarke’s (2021) updated work, the researchers are aware of the importance of reflexive thematic analysis, and that by using the approach inductively, one must keep in mind the theoretical assumptions that have educated their analysis. To engage participants throughout the research process, and to gain a greater level of clarity regarding their experiences, participants were approached to review codes and themes developed throughout the analysis. This approach, referred to as member checking, helps strengthen the credibility of the results and acts as a form of quality control (Birt et al., 2016). Both researchers coded independently, and these codes were then reviewed collaboratively.

Given the nature of the research, focusing on issues of inclusion and exclusion based on participants’ identities, it was acknowledged that this may cause some emotional distress. The researchers both have local and national networks of free support services that participants could access if required. All participants were made aware of their right to withdraw, debriefed if they decided to take part and informed of relevant support agencies, should they have experience psychological or emotional distress during the interview. All participants and venues discussed in this article have been assigned a pseudonym to ensure anonymity of participants and venues. In what follows, we focus our attention on the qualitative data from the interview to explore three key themes developed from participants’ narratives.

## Not Your Space

As participants recruited for this study were diverse in relation to sexual identities, this allowed for clear differences in values and opinions to be established in relation to gender neutral toilets. Whilst most participants in this study appeared to be supportive of gender neutral toilet provision, there appeared to be a difference in comfort levels of accessing and using gender neutral toilets, and also whether they should be the only provision. Lesbian, gay, bisexual, trans, and queer participants generally showed higher levels of comfort with using gender neutral toilets, with no LGBTQ participants expressing any concern about the implementation of gender neutral toilet provision. As Lucy explained:

Instead of renaming the women’s toilets, they should just have gender neutral toilets, get rid of men and women’s toilets altogether and just provide gender neutral toilets. Not just in gay clubs, but everywhere. Being quite butch, I hate using women’s toilets in straight places, I always get looks and feel like I’m being judged

In the quote above, it is clear that Lucy has a preference for using gender neutral toilets and would prefer gender neutral provision to be a standard offer in all spaces. She considers herself to be butch-presenting and therefore experiences the public gaze when utilising sex-segregated facilities. This was a common rhetoric across participants’ narratives, with Patrick, a cisgender gay man, asserting that ‘sex-segregated toilets can be really uncomfortable to use as a gay man, gender neutral toilets are much more comfortable’. Charley, a non-binary individual, noted the personal importance of gender neutral toilet provision in relation to their level of comfort and access to public spaces. This was reiterated by Billy, who is also non-binary when they stated that:

I find it really difficult to go to some public spaces, especially straight ones, because it is just so difficult to go to the toilets, that’s why I like finding a queer space that has gender neutral toilets and I know I won’t experience the same discomfort when I need the toilet.

It is clear that for Billy, the provision of gender neutral toilets has material implications for which spaces they can access safely and comfortably, with Billy noting that queer spaces are more likely to offer this. Whilst gender neutral toilets may be seen as providing more inclusive space for queer people, not all people who occupy queer space feel the same level of comfort with this provision. Whilst many heterosexual participants were supportive of the provision of gender neutral toilets, this did not necessarily translate to a comfortability in accessing them or having *only* gender neutral provision. Francesca, a heterosexual, cisgender woman, who generally approved of gender neutral provision, did not feel comfortable in using them.

While I think it’s great that there are toilets like that to make some people feel comfortable, it shouldn’t be the only option. Like, if I went to a gay club, and they only had gender neutral toilets, I probably wouldn’t go, or I’d be really conscious about what I drink so I didn’t need to go very often, and I wouldn’t go on my own. I’m just not sure that I would be comfortable in them.

In the narrative above, whilst there is a general positivity towards providing gender neutral toilets, it becomes clear that Francesca believes this should be as an addition to sex-segregated toilets, and should not be the only provision. A similar narrative was provided by Harry, also a heterosexual participant, who expressed that ‘they [gender neutral toilets] are great, but I wouldn’t want to use one myself, and I’d probably stop going to the gay village if they only had gender neutral toilets’. What this demonstrates is a level of cisgender privilege. In a heteronormative, cis-normative society in which the needs of heterosexual and cisgender people dominate public discourse and public provision, there appears to be an expectation that queer spaces should actively cater to the needs of cisgender, heterosexual people. The presence of heterosexual, cisgender people in queer spaces has been debated (Hartless, 2019; Moran et al., 2003). Queer spaces have often been understood as a safe space away from heteronormative public spaces, constructed by queer people who historically have been treated as unnatural and polluting the heteronormative public sphere (Hartless, 2019). Therefore, the presence of heterosexual, cisgender people has been considered problematic by some, as a result of the ‘heterosexual gaze’ infiltrating queer spaces (Moran et al., 2003). Notions of infiltration and cisgender privilege became apparent in some participants’ narratives. Michael, a 31-year-old gay man states:

Some of my straight friends have said that they aren’t comfortable with gender neutral toilets in gay clubs, so would only go if there were sex-segregated toilets, but they still want to go there, even when they aren’t invited. They need to realise that that isn’t their space.

There are a number of issues that warrant further discussion, although not all are within the scope of this article, particularly the suggestion that heterosexual, cisgender people should only attend queer spaces with an ‘invite’. However, what is clear from the quote above is that Michael’s friend feel an inalienable right to attend queer spaces, even if they are not comfortable with the toilet provision which challenges heteronormative spatial configurations. This sense of entitlement to access spaces, carved out by queer people to escape the heteronormative public domain, is an example of cisgender privilege. In a cis-normative world, where sex segregation is the status quo, it is clear that some heterosexual, cisgender people feel entitled to access queer spaces, conditionally, providing that the status quo is upheld, and their needs and comfortabilities as heterosexual, cisgender people are catered for. It has been questioned whether ‘straight tourism’ within queer spaces results from heterosexual, cisgender people actually embracing and celebrating queerness, or whether the homonormative arrangements of these spaces allows heterosexual, cisgender people to engage in ‘tolerance’ without questioning and interrogating their own heteronormative standards (Bettani, 2015). This certainly appears to be the case in the excerpt above, in which Michael’s heterosexual friends appear to engage in a ‘performance’ of acceptance, rather than truly embracing and celebrating queer spaces, and the challenges to heteronormative space they present.

## Public Toilets, Queer Needs

As explored earlier, public toilets have been a key discussion point within a range of literature, including gender, sociology and geography. This is due to them being considered the upmost sex-segregated spaces and therefore playing a key role in discussions regarding the enforcement of sex segregation (Doan, 2010; Greed, 2019). Furthermore, some discussions have placed a focus on gender neutral toilets providing a sense of inclusivity and safety, especially for non-binary individuals (Colliver, 2021b). It could also be argued that gender neutral toilets symbolically represent a liminal suspension of gender within these spaces. However, notably, there has been a pushback by some ‘gender critical’ feminists whom one of the things they advocate against is the use of sex-segregated spaces by transgender women (Smythe, 2018). Interestingly, both perspectives follow the assumption that the inclusion of gender neutral toilets has evolved from the wants and needs of the LGBTQ community, as such suggesting that queer needs are of the most, if not only, beneficiary to the inclusion of gender neutral toilets. Similar assumptions have been presented throughout the findings of this study. One participant, Patrick, a gay man, showed his support of gender neutral toilets and their level of importance within queer spaces:

Having gender neutral toilets is really important in gay venues, like more important than other spaces. I think it makes people feel much more comfortable.

A similar perspective was presented by a further participant, David, who noted that ‘[he loved] that there are gender neutral toilets’. Both Patrick and David have shown their positive responses towards the inclusion of gender neutral toilets, and as gay men, it reinforces a sense of acceptance within the LGBTQ community for inclusionary practice. Patrick highlighted how there is a heightened need for gender neutral toilets within LGBTQ spaces. This may be as a result of expected gender non-conformity and expected presence of non-binary people in these spaces. This could reinforce the importance of inclusivity within the LGBTQ community, but it can also highlight the level of ‘othering’ faced by certain marginalised groups and the impact it can have on an individual’s feelings of comfort within public toilets. Furthermore, this reinforces that sex-segregated toilets, of which can exclude non-binary people, are often the norm, and as such, queer safe spaces need to engage in inclusionary practice, such as gender neutral toilets, at a higher volume.

Literature argues that the common perspective is that queer spaces are considered locations that subvert hegemonic gendered expectations (Bailey, 2013), which could leave ‘straight’ spaces not feeling the need to diversify in the way that they are ran. A further set of individuals who have been noted to be affected by this are non-binary individuals. Harry has noted the sex-segregated focus of toilets within mainstream locations is broader than simply the night life environment and is common practice within everyday locations. He refers to a ‘Pizza Express restaurant for example, which has man, woman and that’s it’. Charley, a non-binary individual, has noted how important gender neutral toilets are to them:

Gender neutral toilets are really important to me, being non-binary, I struggle with toilets in public spaces. I always get looked at, like, I feel people are much more judgement in public toilets because they are so used to them being men’s and women’s, and when you don’t visually look like you fit in, people judge you. Queer spaces I usually feel safe in, because I can go to a gender neutral toilet and not feel uncomfortable. I wish that more places had them, like in shopping centres and stuff, because I don’t like using the accessible toilet, it takes away from someone who may need it, and being non-binary isn’t a disability.

Charley has illustrated how gender neutral toilets enable them to enter a space and not feel forced into making a decision that is not fitting with their identity. Literature has recognised how sex-segregated spaces reinforce heteronormative, cis-normative views, and if these expectations are not adhered to, then this can lead to potential judgements being formed (Doan, 2010). Charley has noted how they have experienced this themselves within sex-segregated spaces and how the presence of gender neutral toilets can relieve these negative pressures. Sex-segregated toilets have been referred to as the ultimate location for gender surveillance (Cavanagh, 2010), explaining why Charley is supportive of more inclusivity within common locations, of which is not just limited to the inclusion of accessible toilets. Whilst gender neutral toilets may therefore be seen as increasing the level of accessibility and inclusion for queer people, framing this purely as a ‘queer issue’ has material implications in relation to the politicisation of space, in which gender neutral toilets can therefore be constructed as an unfair demand of a minoritised group (Colliver, 2021c).

In addition to comfort and inclusivity, safety has been a clear factor recognised within this research regarding the reason for why gender neutral toilets are present within queer spaces. When asked about inclusivity, Francesca, a heterosexual woman noted that:

Yeah, I see those toilets [gender neutral] in gay clubs quite a bit, I think it must just be what the community need to make them feel comfortable and safe.

Sex segregation within public toilets has been noted as a particular issue for transgender people, due to the heightened risk to their safety (Faktor, 2011). However, in the quote above, gender neutral toilets are framed as an exclusively ‘queer issue’, in which gender neutral toilet provision functions only to increase the level of comfort of LGBTQ people. Whilst the benefits of gender neutral toilets may extend to a range of people, this does not appear to be considered. In this sense, challenging the status quo of sex-segregated provision becomes associated only with LGBTQ communities. Molly, a heterosexual woman, noted the impact that she believes not having gender neutral toilets present may have on some transgender people:

I think not having the toilets for neutral gender in every club and bar is an issue. Because I think a trans person might feel worried about going somewhere and might not go if that’s not there for them.

The recognition by Molly of the lack of gender neutral toilets within clubs reinforces the narrowing nature of certain spaces for some transgender and non-binary people. Furthermore, she has drawn upon the emotional feelings that may come into play when transgender people are deciding how to navigate their nightlife experience. However, it is important to note that not all transgender people experience difficulties in navigating access to public toilets. This perspective is further developed when considered within the wider sphere of nightlife more broadly. It highlights how within traditional heteronormative, cis-normative spaces, the impact of toilets regarding inclusion is not necessarily catered to. As such, these spaces can unknowingly ostracise particular groups of people and impact the level of accessibility and inclusion for marginalised groups. The level of exclusionary behaviour has been noted by David, a gay man, to not only impact sexuality and gender in the case of some ‘straight clubs’, with David drawing attention to the common use of accessible toilets within these spaces:

I don’t see gender neutral toilets in straight clubs, of course there is sometimes a disabled toilet where sometimes people use it anyway even though they don’t need to, but that’s the closest you normally get in straight places.

It is worth noting that accessible toilets may also be used by those with hidden disabilities and that the stereotype around the term disabled often excludes those with hidden disabilities or other impairments (Hanson, 2004). However, with this being said, the volume of use that David appears to notice would suggest that the use of these accessible toilets is not always legitimate. As such, marginalisation through toilets impacts those with visible and hidden disabilities, as well as other marginalised groups. This perspective would suggest that even when procedures for inclusion are put in place within traditionally heteronormative spaces, they are not necessarily managed to ensure they are supporting the needs of those who have been marginalised.

By David mentioning that accessible toilets are ‘the closest you normally get’, suggests that in terms of toilet inclusion, accessible toilets are most likely to be the only non-sex-segregated provision within traditionally heteronormative nightlife environments, in turn suggesting that gender neutral toilets are more of a rarity within ‘straight’ clubs. This perspective was further developed by Harry, a heterosexual man who has had several experiences visiting both traditionally heteronormative and LGBTQ venues:

.. I don’t think, drawing upon what I talked about in terms of misogyny and just this general toxic masculinity, I don’t think Vicarage Street provides much of a diverse environment for the LGBT community as [a Gay Village within the West Midlands] does because, I’ve never seen any signage, you know, things like gender neutral toilets for example. I might be completely wrong… there doesn’t appear to be as much inclusive policies in place just to support LGBTQ individuals as opposed to what they have in the [a Gay Village within the West Midlands]. So, yeah, Vicarage Street is diverse in terms of ethnicity but is not as diverse in terms of sexuality.

In addition to the somewhat level of inclusivity that has been mentioned previously in regard to the implementation of accessible toilets, albeit not necessarily respected by all consumers, ethnicity is a further area whereby diversity is considered within heteronormative spaces. However, Harry has highlighted that mainstream locations will tend to follow more heteronormative approaches, therefore reducing the desire to provide a more inclusive or safe environment based on LGBTQ needs. In his discussion of inclusion of LGBTQ people, Harry makes explicit reference to the lack of gender neutral toilets, again positioning gender neutral toilets as an exclusively ‘queer need’, negating the potential benefits they may provide to a range of people.

Gender neutral toilets have been considered inclusive, safe spaces, for those individuals who do not meet heteronormative and cisgender expectations (Colliver, 2021b). Alongside this, however, is the perspective that the need or desire for gender neutral toilets is explicitly a ‘queer’ issue. Many participants have not only highlighted the likelihood of gender neutral toilets appearing at queer spaces, but also the rarity of them appearing in heteronormative spaces. Although inclusion in terms of disability and ethnicity has been identified within heteronormative spaces, little inclusion on the basis of sexuality and non-binary individuals has been noted within this research. Queer spaces are more commonly known for requiring a strong move towards inclusion based on LGBTQ needs, of which gender neutral toilets support. This inclusion is supportive and beneficial to many; however, it appears to have negated the need for progression to occur outside of ‘queer spaces’.

## It’s a Man’s World

One of the dominant themes that was developed from the data and underpinned a significant amount of participants’ narratives, related to the provision of toilets for men in queer spaces. In this sense, toilet provision within queer spaces was conceptualised as being one of the ways in which queer spaces are designed to meet the needs of men, and this was often seen as being at the expense of other groups. This was an issue in venues that provided sex-segregated toilets and also in venue’s that provided a gender neutral toilet alongside sex-segregated toilets. In venues which only offered gender neutral toilets, this was not seen as a problem. The sense of centring men was often as a result of the physical location of sex-segregated toilets, with men’s convenience seen as a priority.

Often, participants noted that when gender neutral toilets were available, these were often not custom-designed, but rather were formerly women’s toilets that had been re-purposed or simply had signage changed to indicate they were now gender neutral toilets. As Lucy, a 30-year old lesbian woman described:

It’s really shitty, because while I think gender neutral toilets are a good idea… in Cameo they have just changed the sign on the women’s toilets, so you now have a gender neutral toilet right next door to a men’s toilet. I get that it’s easier because there aren’t urinals in the women’s toilet, but surely it couldn’t be that much work to just get rid of the urinals. It seems like all the gender neutral toilets I see are just women’s toilets that have been renamed.

Whilst it is clear that Lucy appreciates the provision of gender neutral toilets, it is also seen as being at the expense of toilet provision for women. This is unsurprising, as Greed (1995) found that in relation to public toilets the convenience afforded to men was significantly higher than that afforded to women and this was evident through the provision of two-thirds more public facilities. This also centres the needs of men, as it increases the provision of toilet facilities for men, whilst there may be an increased flow of traffic into the gender neutral toilets, including men, which may cause longer queue times and less provision for women. What became clear through participants’ narratives was a general approval of gender neutral toilets being provided, but a desire for these to be custom-built, rather than simply changing the signage on existing women’s toilets.

The provision of gender neutral toilets was also seen to be symbolic and political within queer spaces. Whilst queer movements have often been inherently political and oppositional to dominant, heteronormative culture, this also transcends into queer spaces. As Charley, an 18-year old queer, non-binary individual argues:

Gender neutral toilets should be everywhere, but they have to start in queer spaces. Gender neutral toilets go against the grain. It is great to see them in queer spaces, it makes it much more comfortable for me to just do something as simple as go to the toilet. Queer people, queer politics and queer spaces have to lead the way in challenging the status-quo, in changed the binary way the world is structured. If we can’t get it right, or get it done in our own spaces, we will never get the mainstream to change for us.

In the quote above, Charley draws upon the politically symbolic power of offering gender neutral toilets. In a patriarchal society, in which public spaces are traditionally designed for, and dominated by men (Thompson, 1993), the provision of gender neutral toilets may be understood as deviating from patriarchal expectations around provision and convenience and challenging the status quo. Therefore, the value of gender neutral toilets is two-fold, in both challenging structural inequalities at a symbolic level, whilst improving inclusion and accessibility on a practical level. However, it became clear throughout the interviews that challenging the status quo was not welcomed by all participants, and this became evident primarily in relation to differences in sexualities.

In venues where toilet provision is split over two floors, female participants noted that toilet provision for women was often located further away than toilet provision for men. As Jennifer, a 29-year-old heterosexual woman explains:

The first bit that you walk into there’s, like, bathrooms, but the bathrooms were just for men, and if I needed to go to the bathroom I’d have to go upstairs, and usually if you go upstairs, you would think there would be, like, another space to, like, dance and enjoy yourself, but no, it was just the bathroom. And what I found really strange was the underground had gender neutral bathrooms.

There are a number of points worth discussion in the narrative provided by Jennifer. Firstly, there is something to be said about the physical location of sex-segregated toilets, which was also picked up by other female participants. Issues of male convenience are not a new concept, and it has been argued that public toilet provision has historically met the needs of men, rather than women (Cavanagh & Ware, 1990). Similarly, issues of male convenience are often drawn upon in online discourse around the implementation of gender neutral toilets, with the gender neutral toilets often being understood as a loss of men’s convenience (Colliver, 2021c). This was reiterated by Francesca, a 22-year-old heterosexual woman, who noted:

I think most of the gay places I have been to when there are toilets on different floors usually have the men’s as soon as you walk in and then the women’s are hidden somewhere else, usually up or down a flight of stairs, or right at the back of a venue.

This may be because of queer spaces being male-centric, with gay and queer men outnumbering gay and queer women. Indeed, in research exploring Manchester’s gay village, Pritchard et al. (2002, 105) note that the exclusion of women, and in particular, lesbian women, results from the ‘homo-patriarchic power dialectics’ and a ‘more established gay male community’ that is not inclusive of women. In this sense, the spatial positioning of toilets within queer spaces reaffirms a male-centric culture. It also symbolically positions the needs of everyone other than men as ‘out of the way’.

The second point that is worth further discussion is the spatial positioning of gender neutral toilets ‘underground’. The venue that Jennifer is describing has both a ground floor and a ‘basement bar’. Whilst toilets for men are positioned on the ground floor, and the toilets for women are located upstairs on the first floor, the basement bar offers gender neutral toilets. The gender neutral toilets in the basement bar are made up of three small cubicles with a shared area with two basins for hand-washing. Given the multiple offers of toilet provision within this venue, the positioning of gender neutral toilets underground has symbolic value. In some ways, this positions gender neutral toilets as undesirable and simultaneously unseeable to the homonormative, mainstream clientele who occupy the ground floor. This may therefore create issues in relation to inclusion and a sense of belonging to those who may actively seek out gender neutral toilets to use.

## Conclusion

Access to public toilets has become a core feature of public debate surrounding transgender equality and inclusion. In this article, we have interrogated the ways in which people engage with and access toilet provision within queer spaces in the West Midlands. Whilst existing research has focused on toilet provision within heteronormative public spaces and addressed issues of unequal gendered toilet provision, we have extended this knowledge by addressing both the practical and symbolic value on gender neutral toilet provision within queer spaces. Resultantly, there are a number of key policy implications that can help advance access and inclusion within social spaces.

Central to this article are three key claims. Firstly, we have argued that gender neutral toilet provision is often framed as a ‘queer need’, understood to solely benefit queer communities, with no acknowledgement of the broader benefits that this type of provision may have for other populations. Although there have been significant legislative changes to service provision, with a particular focus on equalities legislation, working at purely a legislative level, without structural change is unlikely to increase inclusion for marginalised groups. Whilst many LGBTQ people acknowledge the benefit of gender neutral toilets in queer spaces, in relation to inclusion and accessibility, positioning gender neutral toilets as an exclusively queer need has material implications for widening access and inclusion within heteronormative public spaces. This framework for understanding gender neutral toilet provision is regularly drawn upon to construct queer communities as demanding, boisterous, and also feeds into claims around heterosexual, cisgender ‘victimhood’, in which dominant communities claim a victim position, victimised by radical, marginalised communities (Colliver, 2021c). It is therefore key to highlight the potential benefits that gender neutral toilets have for a range of communities and also how they may begin to address gendered toilet parity. It also emphasises the importance of providing gender neutral facilities within wider ‘heteronormative’ spaces, in order to challenge structural and cultural norms that position queer communities as outsiders.

Secondly, we have claimed that queer spaces are often seen as catering to the needs of cisgender men, through the spatial positioning of sex-segregated toilets within queer spaces. The symbolic nature of the spatial positioning of women and gender neutral toilets is powerful and positions the needs of those other than men as ‘out of the way’. We therefore argue that whilst queer spaces have traditionally been considered to challenge heteronormative structures, the provision of sex-segregated toilets centres the needs of men and therefore reinforces patriarchal social structures in which men’s convenience is paramount. The provision of gender neutral toilets within queer spaces, therefore, has practical value in relation to accessibility and inclusion, but also challenges the status quo. There are also policy implications for the design and spatial positioning of sex-segregated toilets which, if utilised, can offer greater inclusion and comfort for women, who have historically experienced toilet inequality.

Finally, we have made the claim that in a heteronormative, cis-normative society, it is not the work of queer people to make queer spaces accessible for heterosexual, cisgender people. Indeed, in a society dominated by heterosexual, cisgender people in which their needs and comfortabilities are often central to social life, often at the expense of marginalised groups, it is the job of queer spaces to provide a haven away from these structures and to challenge the foundations that maintain the status quo. In order for queer spaces to maintain their oppositional foundations, the needs of queer people must continue to be centred, and heterosexual, cisgender engagement and occupation of these spaces must be celebratory, and not performative.

## Data Availability

The data used in this article are not stored in an online repository.

## Footnotes

1 A ‘gay village’ is a geographical area in which venues within the community known as LGBTQ + spaces cluster around.
